# Visual Noise Effect on Contour Integration and Gaze Allocation in Autism Spectrum Disorder

**DOI:** 10.3389/fnins.2021.623663

**Published:** 2021-02-09

**Authors:** Milena Slavcheva Mihaylova, Nadejda Bogdanova Bocheva, Tsvetalin Totev Totev, Svetla Nikolaeva Staykova

**Affiliations:** ^1^Department of Sensory Neurobiology, Institute of Neurobiology, Bulgarian Academy of Sciences, Sofia, Bulgaria; ^2^Department of Psychiatry and Medical Psychology, Medical University of Sofia, Sofia, Bulgaria

**Keywords:** contour integration, visual perception, ASD, neural noise, external noise, eye movements

## Abstract

Contradictory results have been obtained in the studies that compare contour integration abilities in Autism Spectrum Disorders (ASDs) and typically developing individuals. The present study aimed to explore the limiting factors of contour integration ability in ASD and verify the role of the external visual noise by a combination of psychophysical and eye-tracking approaches. To this aim, 24 children and adolescents with ASD and 32 age-matched participants with typical development had to detect the presence of contour embedded among similar Gabor elements in a Yes/No procedure. The results obtained showed that the responses in the group with ASD were not only less accurate but also were significantly slower compared to the control group at all noise levels. The detection performance depended on the group differences in addition to the effect of the intellectual functioning of the participants from both groups. The comparison of the agreement and accuracy of the responses in the double-pass experiment showed that the results of the participants with ASD are more affected by the increase of the external noise. It turned out that the internal noise depends on the level of the added external noise: the difference between the two groups was non-significant at the low external noise and significant at the high external noise. In accordance with the psychophysical results, the eye-tracking data indicated a larger gaze allocation area in the group with autism. These findings may imply higher positional uncertainty in ASD due to the inability to maintain the information of the contour location from previous presentations and interference from noise elements in the contour vicinity. Psychophysical and eye-tracking data suggest lower efficiency in using stimulus information in the ASD group that could be caused by fixation instability and noisy and unstable perceptual template that affects noise filtering.

## Introduction

Atypical processing of low-level sensory information has been reported in Autism Spectrum Disorder (ASD) (Dakin and Frith, 2005; Simmons et al., 2009) in addition to impaired social and higher-level cognitive abilities, restricted and repetitive behaviors. The significance of sensory symptoms, like abnormal reactivity to sensory stimuli manifested as either hyper- or hypo-sensitivity is emphasized by their inclusion in the Diagnostic and Statistical Manual of Mental Disorders (American Psychiatric Association [APA], 2013).

One of the most notable examples of altered perception in ASD is the compromised processing of social stimuli such as faces. Along with the assumption that the impairment derives from a pervasive problem in social interaction and motivation, several studies are suggesting that the visual perceptual alterations may contribute to the difficulty with face processing as well (reviewed by Behrmann et al., 2006). A possible mechanism of the suboptimal face processing could be connected to the well-described diminished ability in ASD to group local visual elements that are presented in different parts of an image into a global percept despite the enhanced processing of visual details (Behrmann et al., 2006; Happé and Frith, 2006).

Different neurophysiological studies explored the question of how local signals are integrated across space to generate global percepts. The data obtained are interpreted as evidence that horizontal, feedforward, and feedback connections between neurons in the visual system, particularly in the primary visual cortex (V1), are responsible for the visual integration (e.g., Kapadia et al., 2000; Angelucci et al., 2002; Nurminen and Angelucci, 2014).

At the psychophysical level, the ability to group or integrate local visual elements has been often explored by contour integration studies that involve the detection of a contour consisting of Gabor elements embedded among a background of randomly oriented Gabors (e.g., Field et al., 1993; Jachim et al., 2015). The target contours could represent a single line, named as an open contour (Field et al., 1993), or a predetermined shape, closed contour (Jachim et al., 2015). Kovács and Julesz (1993) first reported that it is much easier to detect closed than open contours and their finding was repeated in later studies (Mathes and Fahle, 2007; Gerhardstein et al., 2012; Jachim et al., 2015). In order to explain this facilitated detection, it was suggested that in an early vision a synergetic process exists (Kovács and Julesz, 1993) or a separate mechanism that is sensitive to the detection of closed contours (Gerhardstein et al., 2012). Mathes and Fahle (2007) suggested that closed contour facilitation may occur at both early visual areas which are responsible for local orientation information processing and at higher visual areas (the lateral occipital complex) which process the global shape of the contour.

An important factor that determines the contour integration ability is the alignment of the elements along “the path” (Field et al., 1993). The detection of contours is diminished when the relative orientation or jitter of adjacent Gabor elements is increased (Field et al., 1993; Jachim et al., 2015).

The results, obtained in the studies that compare contour integration abilities in ASD and typical development (TD), are contradictory. Del Viva et al. (2006) found similar spatial integration performance between children with ASD and TD when detecting a circle embedded in noise. The elements of both the circle and the noise were Gabor patches presented for 1 s on a computerized display. The authors interpreted these findings as a demonstration of preserved early perceptual integration. Kemner et al. (2007) applied a card-based version of the contour integration task with closed contour stimuli and over a second-long presentation time. They similarly found normal performance in the group with a pervasive developmental disorder compared to the control group.

Contrary to these results, contour integration ability was challenged in later studies. By applying an electrophysiological paradigm Pei et al. (2009) searched for neural correlates of the local visual signals integration in a group of low functioning children with ASD and an age-matched control group. The stimuli were Gabor elements that alternated every 500 ms forming circular contours or random patterns. It was found that the 3rd harmonic response was absent in the group with autism in contrast to the control group. The authors interpreted this finding as a neural correlate of highly specific deficiencies that could be connected to some deficits in ASD like face avoidance or reading abnormalities. Evers et al. (2014) compared the identification of gradually appearing contours by aligning local Gabor elements toward randomly oriented Gabor elements. The result showed that the identification performance of the children with ASD was slower and less accurate than that of the controls with TD, especially when more complex contours were shown. The results were interpreted as evidence of an impaired relationship between local-global and bottom-up-top-down processes in autism. Hadad et al. (2019) also reported slower and less accurate responses in the ASD group than in the TD group in identifying contours based on everyday objects. However, the authors suggested that the group differences could indicate known differences between the groups in response times and general tolerance to noise, rather than in the mechanism of spatial integration.

It seems that at least several factors could be responsible for the contradictory results between the different studies on contour integration ability in autism. Jachim et al. (2015) suggested that the peculiarities of the atypical contour integration in ASD became obvious mostly in cases of object identification instead of detection of simple shapes. In their study, Jachim et al. (2015) found less improvement in contour detection between open and closed contours in adults with ASD compared to a control group with TD, although there was not a group difference with either open or closed stimuli. In contrast to the last finding in the newest study on this topic (Gowen et al., 2020) better perceptual performance for ASD compared to the TD group was observed for the open stimulus in addition to the replication of the reduced closure effect. As possible explanations, the authors discussed several possibilities: the involvement of autistic participants with an enhanced perceptual ability, the difference in the number of Gabor elements in the open contour, as well as the possibility for more eye movements toward the contour made from the autistic group and thus improving the contour detection because the central instead of the peripheral location improves contour integration (e.g., Hess and Dakin, 1997; Nugent et al., 2003). However, Gowen et al. (2020) suggested that eye movements could hardly influence their results since the deviations from the fixation point greater than 2.5° from the center were removed and there was no improvement in performance between short and long stimulus duration.

Long stimulus duration is indicated as a possible factor that could hide any differences in contour integration since people with ASD may need more time to discern the figure (Jachim et al., 2015). Based on a meta-analysis Van der Hallen et al. (2015) concluded that global-order perception is slower in ASD than in TD. However, the results of Gowen et al. (2020) showed a similar reduced closure effect in the ASD group compared to TD at short (150 ms) and long (500 ms) stimulus presentation times, thus rejecting the possible role of slower global processing. Nevertheless, there are still considerations that participants with ASD could apply a different strategy in contour integration tasks (reviewed by Jachim et al., 2015) or in face recognition tasks (e.g., Deruelle et al., 2004; Ashwin et al., 2006), especially at longer stimulus durations.

Generally speaking, it has been assumed that two types of determinants govern human signal-detection responses: external and internal (e.g., Burgess and Colborne, 1988). While external determinants are connected to the nature of the signal, the noise, and the task, variability in the internal determinants is commonly attributed to internal noise. Internal noise influences the nervous system at each level starting from the perception of sensory signals to the generation of motor responses (Faisal et al., 2008) and has been reported in sensory and motor systems of individuals with autism (Simmons et al., 2009; Dinstein et al., 2015). Higher neural variability in visual, somatosensory, and auditory modality was demonstrated in functional magnetic resonance imaging (fMRI) studies by poor evoked response reliability when comparing cortical response amplitude and consistency across trials (Dinstein et al., 2012) or by greater intra-individual variability in the sensory-evoked fMRI responses (Haigh et al., 2015). In support of the assumption about the increased neural noise in ASD are also results from electrophysiological studies. Milne (2011) observed significantly greater intra-participant electroencephalogram (EEG) variability and lower inter-trial α-band phase coherence in ASD individuals than in neuro-typical matched controls. Weinger et al. (2014) reported lower signal-to-noise ratios and deficits in low-contrast responses at the stimulus frequency of 12.5 Hz in the ASD group compared to the TD group. Increased inter-trial variability in ASD that resulted in reduced P100 amplitude was recently described by Kovarski et al. (2019).

Psychophysical features in ASD such as high visual motion coherence thresholds (Milne et al., 2002) and broad tuning of auditory filters (Plaisted et al., 2003) could be explained by high levels of noise in neural networks as suggested by Baron-Cohen and Belmonte (2005). The signal-to-noise ratio could be reduced if a network is overconnected and sensory inputs evoke atypically large activations for both attended and unattended stimuli resulting in an overall unselective increase of activation (Belmonte et al., 2004). However, the results of other studies (Brock et al., 2002; Just et al., 2004) imply diminished connectivity. Baron-Cohen and Belmonte (2005) suggested that this contradiction could be explained by the possibility that the high connectivity within local networks could develop together with atypically low computational connectivity with other regions.

Excessively high levels of neuronal noise could be generated at both the neural network level and at the single-cell level. Increased inner noise may result from high variability of neuronal activity in peripheral receptors (Schneeweis and Schnapf, 1999, 2000; Faisal et al., 2008), or synaptic transmission variability due to the probabilistic nature of the neurotransmitter release and the variable timing and amplitude of the post-synaptic response (Ribrault et al., 2011). Mechanisms that target excitatory and inhibitory synapses, and mechanisms that target intrinsic neuronal excitability support the balance between excitation and inhibition that could be probably compromised in autism (Turrigiano, 2011). Persico and Bourgeron (2006) reviewed genetic, epigenetic, and environmental factors that could contribute to autism. The authors suggested several major pathways that are concerned in ASD pathogenesis: altered cell migration, the glutamate–GABA equilibrium, synapse formation and maintenance, as well as dendritic morphology. Single-neuron sensory responses depend on the states of their neural networks and changes in levels of attention and excitement (Fontanini and Katz, 2008). At the neural network level, variability can be increased due to disturbances of excitation/inhibition balance through increased levels of excitatory inputs (Rubenstein and Merzenich, 2003; Trakoshis et al., 2020) as well as by continuous interaction and competition between functional brain networks (Kelly et al., 2008). Network inefficiencies could be connected to deficits in connectivity related to low-level processing and could potentially affect higher-level cognitive processes and social behavior (Lewis et al., 2017).

However, it should be noted that the question of the higher internal noise in ASD is still disputable. Butler et al. (2017) observed similar levels of variability in visual and somatosensory evoked EEG using high-density mapping in individuals with ASD and TD. The comparison of the magnetoencephalographic response to passive tactile stimulation failed to show higher variability in the ASD group than in the group with TD (Coskun et al., 2009). A psychophysical study on motion integration applying the equivalent noise approach, which uses different quantities of external noise added to the stimulus, (Manning et al., 2017) revealed enlarged sampling in children with ASD for motion information but no convincing evidence for abnormal levels of internal noise. Davis and Plaisted-Grant (2015) suggested that symptoms of ASD could be explained by reduced instead of increased endogenous noise, which is probably a function of abnormal brainstem activation. Low internal noise would lead to increased detection and discrimination in ASD. However, a low-noise brain will not gain benefits of noise in neural networks and may fail to generalize learning from one context or stimulus to others; become “stuck” in a certain mode of thought or action; may not respond reliably to a stimulus across presentations.

Concerning the external determinants of the signal-detection response, it should be noted that in most contour integration studies, external noise is inherent to the stimuli since the target contour is constructed from elements positioned among many similar “noise” elements. The physical randomness in the external environment could induce perceptual variability (Bialek, 1987). Moreover, Osborne et al. (2005) supposed that even the variability in movements could result from errors in the sensory estimates of the external parameters defining the appropriate action rather than by noise in the motor system itself. The irrelevant noise in the sensory signal is usually excluded through a process of external noise filtering by an appropriate perceptual template, thus diminishing the negative effects of added noise (Lu and Dosher, 2008; Park et al., 2017). The ability to filter the noisy signals would maintain our perception stable, while suboptimal external noise filtering would reduce perceptual efficiency.

The ability to filter the noisy signals is diminished in ASD (Park et al., 2017). Manning et al. (2015) suggested that segregation of signal from noise could be a limiting factor for individuals with autism across a range of motion processing tasks. Children with autism showed enhanced motion integration compared to typical children, but similar performance in the motion coherence task, which requires reporting the direction of coherently moving dots among randomly moving noise dots. These results were interpreted as an implication that the motion coherence thresholds in autism may be affected by diminished discrimination of signal from noise. The authors suggested that seemingly advantageous increased integration may lead to feelings of “sensory overload” in children with ASD. Sanchez-Marin and Padilla-Medina (2008) found that children with autism detected a simple visual signal, still or in motion, embedded in Gaussian noise, significantly worse than children with TD. The authors concluded that this result is not connected to a limited ability to detect simple visual stimuli in autism because the stimuli used in their study were not easy to detect, even for TD children. Most probably, the overwhelmed or disturbed children’s ability to process the visual information due to the background noise and motion was responsible for the results. Except for the additive noise, the induced internal noise (Burgess and Colborne, 1988) proportional to the external-noise spectral density could also limit behavioral performance. It is possible that the induced internal noise increases more strongly for the observers with ASD than for observers with TD, and this could lead to anomalous processing of the detected information (Sanchez-Marin and Padilla-Medina, 2008). Zaidel et al. (2015) found that the addition of stimulus noise to visual motion through a cloud of dots affected significantly more the perception of adolescents with ASD than controls despite that the results of both groups were similar without noise. The authors interpreted these results as increased sensitivity to sensory noise and less use of prior knowledge in ASD.

The perceptual efficiency could be reduced by both poorer external noise filtering and excessive neural variability levels referred to as neural or inner noise (Park et al., 2017). Results of Park et al. (2017) demonstrated that both factors are affected in ASD: the internal noise is elevated, and the external noise filtering is diminished. A complicating factor is the difficulty to separate the effects of diminished external noise filtering and increased internal neural noise. External sensory stimuli being naturally noisy could influence the internal noise and could increase trial-to-trial variability at the first stage of perception during the processes of conversion into a chemical or mechanical signal as well as during the following processes of amplification and transduction of the sensory signal and conversion it into an electrical impulse (Faisal et al., 2008).

The aim of the present study was to explore the limiting factors in contour integration processing in ASD. We tried to evaluate the potential role of elevated internal noise and a noisy or variable perceptual template for contour detection using psychophysical methods and eye-movements recording. To achieve this goal, we suggested a stimulation that differs in several aspects from the typical studies on contour integration. A significant difference is that while in the other studies, the background elements are distributed pseudo-randomly on a square grid, in our study, all elements are positioned precisely at the intersection points of a regular hexagonal grid. Therefore, their centroids are aligned with the grid, and no positional information distinguishes the contour elements from the background noise. The observers had to detect a tilted straight contour aligned with a virtual line from the grid among randomly oriented similar elements. The position of the contour (when present) was fixed. We varied the contour strength by changing the orientation of the contour elements by variable amount keeping the mean contour orientation the same but altering the orientational variability. The increased orientation variance represents the external noise added to the contour. This manipulation effectively changes the similarity between the contour and background elements. We limited the stimulus presentation to 200 ms to minimize the possible impact of uncontrolled eye movements and to restrict the possibility of searching behavior. However, we registered the observers’ eye positions during stimulus presentation to obtain information on whether their gaze positions vary with the stimulus characteristics.

We presented the stimuli with the same orientational variability in blocks. This would allow the observers to obtain a proper template for each contour strength. While the observers could not change their gaze allocation during the short stimulus presentation, they could have moved their eyes during the fore-period due to either fixation instability or differences in the template. The fixation instability should be independent of the stimulus while the stimulus-dependent gaze shifts and their variability can provide a measure of template stability. In addition, we used the double-pass paradigm (Burgess and Colborne, 1988) at two noise levels – low and high. This paradigm is regarded as the most appropriate for evaluating the factors limiting human performance. The methodology allows partitioning the behavioral variability in correlated and uncorrelated factors. The correlated factors are related to the stimulus variability, while the uncorrelated ones are due to the internal noise that randomly changes. The double-pass paradigm consists of repeating the stimulus sequence and comparing the agreement between the responses to the same stimuli in the two repeats and the accuracy of performance. If no internal noise limits the performance the responses in the two repeats should be the same, whereas the accuracy will be determined by the stimulus variability. At low levels of stimulus variability (low levels of external noise), the performance will be limited by the additive internal noise. At high levels of external noise, the contribution of the additive internal noise becomes negligible and the behavioral performance is limited by stimulus-dependent (multiplicative) noise or by suboptimal computations like missing important stimulus features or using irrelevant stimulus characteristics i.e., the irrelevant information is not filtered. The double-pass paradigm allows the evaluation of the ratio of the internal to external noise. Therefore, it permits comparisons of the internal noise levels between the ASD and TD groups at the same external noise level.

We tried to restrict the confounding effect of some of the factors mentioned above. To avoid an influence from hierarchically higher areas like the lateral occipital complex (Murray et al., 2002; Gilad et al., 2013), we decided to use open contours instead of closed contour stimuli. We tried to make the participants’ task as simple as possible to prevent the task difficulty effect on the results. To prevent the participants with ASD from using a different strategy to determine contour presence or absence, the stimulus duration in our experiments was limited to a short presentation time. To cover a representative part of the autistic spectrum, we tried to include in our study children and adolescents with a wide range of IQ and different proximity to the ASD cut-off as calculated by ADI-R.

We expect that if the participants with ASD have higher levels of additive internal noise or could not filter the background noise, their performance would be worse than that of the participants with TD, even when no external noise is added to the contour. If participants with ASD have higher stimulus-dependent or induced noise, they will show reduced agreement between the responses in the two repeats at the higher level of external noise in the double-pass experiment. If the response time in ASD varies in a stimulus-dependent manner, this will imply that the potential differences between the ASD group and the TD group are not connected only to the preparation and the execution of the motor response. Stimulus-dependent changes in the response time may reflect the different time needed for stimulus encoding at the different levels of external noise or the difference in the rate of evidence accumulation for a particular response choice due to task difficulty changes. If the gaze positions vary with the added external noise, this might be regarded as a noisy or variable template for contour detection at different noise levels.

## Materials and Methods

### Participants

Sixty children and adolescents participated in the study: 28 in the ASD group (4 were later excluded from the analysis) and 32 in the TD group. The participants were recruited via the Sofia Center for Social Rehabilitation and Integration–autism spectrum priority, the Regional Center for Support of the Inclusive Education Process-Sofia-city, Regional Department of Education–Sofia city and through community organizations, parental associations, and professionals (psychologists, speech therapists, child psychiatrists, etc.).

Brief interviews and a developmental questionnaire (filled by parents) ensured that none of the participants in the study have a history of previous neurological or psychiatric disorder (other than ASD in the experimental group), head trauma, current psychoactive medication, and the presence of a visual impairment that could interfere with the performance of tasks. No learning or language difficulties were reported for the TD group. Wechsler Intelligence Scale for Children–Fourth Bulgarian Edition (WISC–IV BG, 2015; Wechsler, 2003) was administrated for both groups, resulting in Verbal Comprehension Index (VCI), Perceptual Reasoning Index (PRI), Working Memory Index (WMI), Processing Speed Index (PSI), and Full-Scale IQ (FSIQ) (see Table 1).

**TABLE 1:** Sample characteristics.

	**ASD group (*N* = 24)**	**TD group (*N* = 32)**
*N* (male/female)	24 (16/8)	32 (24/8)
Age Mean ± SD [range] in years	11.6 ± 2.4 [8–16]	11.6 ± 2.4 [8–16]
WISC-IV (Mean ± SD [range])		
VCI	81.62 ± 18.42 [45–124]	105.15 ± 11.11 [85–142]
PRI	90.00 ± 22.75 [50–136]	99.46 ± 13.61 [76–129]
WMI	86.50 ± 18.22 [59–123]	103.43 ± 10.97 [77–123]
PSI	84.87 ± 16.82 [55–139]	99.18 ± 12.52 [76–124]
FSIQ	84.04 ± 17.24 [59–122]	102.28 ± 13.30 [80–141]

At first, the ASD group consisted of 28 children and adolescents, 4 of whom were unable to perform the experimental task adequately and their data were excluded from the analysis. Thus, the final sample included 24 participants with ASD (16 boys, and 8 girls; mean ± SD [range] age = 11.6 ± 2.4 [8–16] years). All of them had already been diagnosed with a pervasive developmental disorder (including Autism, Asperger’s syndrome, and ASD) according to ICD-10 (International Statistical Classification of Diseases and Related Health Problems 10th Revision, 1990) criteria. For the study, the diagnosis was confirmed by an experienced clinical psychologist using the Autism Diagnostic Interview-Revised (ADI-R) (Lord et al., 1994; Rutter et al., 2003) and a review of their most recent developmental and medical reports. The ADI-R is a detailed semi-structured interview of parents about their child’s developmental history and autism symptoms that yield ratings for qualitative abnormalities in reciprocal social interaction (Score A), language, and communication (Score B), restricted, repetitive, and stereotyped patterns of behaviors (Score C), and abnormality of development (Score D). The scoring algorithm is similar to the diagnostic criteria of ICD-10 and DSM-IV. It is comprised of 93 items, 42 of which can be ranked into the following four scores with the respective cutoff values for diagnostic purposes: Score A- 10; Score B- verbal 8; Score C- 3; and Score D- 1. All participants in the experimental cohort of the study have results that meet the requirement the child must score above the cut-off level in each of the three domains and exhibit some abnormality in at least one area by age of 36 months, and they were classified as patients with autism according to their scores from ADI-R (see Table 2).

**TABLE 2:** ADI-R domain-specific scores.

	**ASD group (*N* = 24)**
ADI-R (Mean ± SD [range])	
Score A Qualitative Abnormalities in Reciprocal Social Interaction	26.16 ± 4.47 [11–30]
Score B Qualitative Abnormalities in Communication	19.66 ± 4.21[9–24]
Score C Restricted, Repetitive, and stereotyped behavior	7.12 ± 2.77 [2–12]
Score D Abnormality of Development Evident at or Before 36 Months	4.33 ± 0.96 [2–5]

Thirty-two typically developing children and adolescents, matched for age and sex to the ASD group, formed the control sample (24 boys and 8 girls; mean ± SD [range] age = 11.4 ± 2.3 [8–16] years). They were recruited from local schools and attended regular school classes at expected grade levels. The parents confirmed in writing that their child did not have a history of any mental or neurological diagnosis.

As expected, an independent-samples *t*-test confirmed that the two groups did not differ in age: *t*(54) = 0.324, *p* = 0.747, and sex *t*(54) = −0.674, *p* = 0.503. Although the groups with ASD and TD were carefully matched in terms of age and sex, matching IQ score was a challenge as we wanted to include in the study as wide as possible group of participants from the autism spectrum, that would result in different levels of intellectual functioning, and the difference in WISC score was expected: FSIQ *t*(54) = −4.471, *p* < 0.05, VCI *t*(54) = −5.934, *p* < 0.05, WMI *t*(54) = −4.322, *p* < 0.05, and PSI *t*(54) = −3.652, *p* < 0.05. There was no significant difference in mean PRI score between ASD and TD groups *t*(54) = −4.471, *p* > 0.05.

Five of the participants in each group dominantly used the left hand. All participants had normal or corrected-to-normal near and far visual acuity, measured by Rosenbaum Pocket Vision Screener and Tumbling “E” Test, respectively at 35.6 cm and 3 m. All had 1200″ stereo acuity measured by Lang stereo test and normal contrast sensitivity measured by Hamilton-Veale Contrast Sensitivity Test.

### Stimuli and Procedure

The stimuli were generated by a custom software and presented on an EIZO CS230 23″ monitor with a vertical refresh rate of 60 Hz and a screen resolution of 1920 × 1080 pixels. The stimulation field had a mean display luminance of 18 cd/m^2^ and a size of 22.5 × 40° (ratio 16/9). The monitor’s default settings and calibration were checked and controlled by X-Rite i1 Eye-One Monitor Calibrator. Custom software written in C + + was used to generate the stimuli by an OpenGL video card and to control the experiment.

**FIGURE 1:**
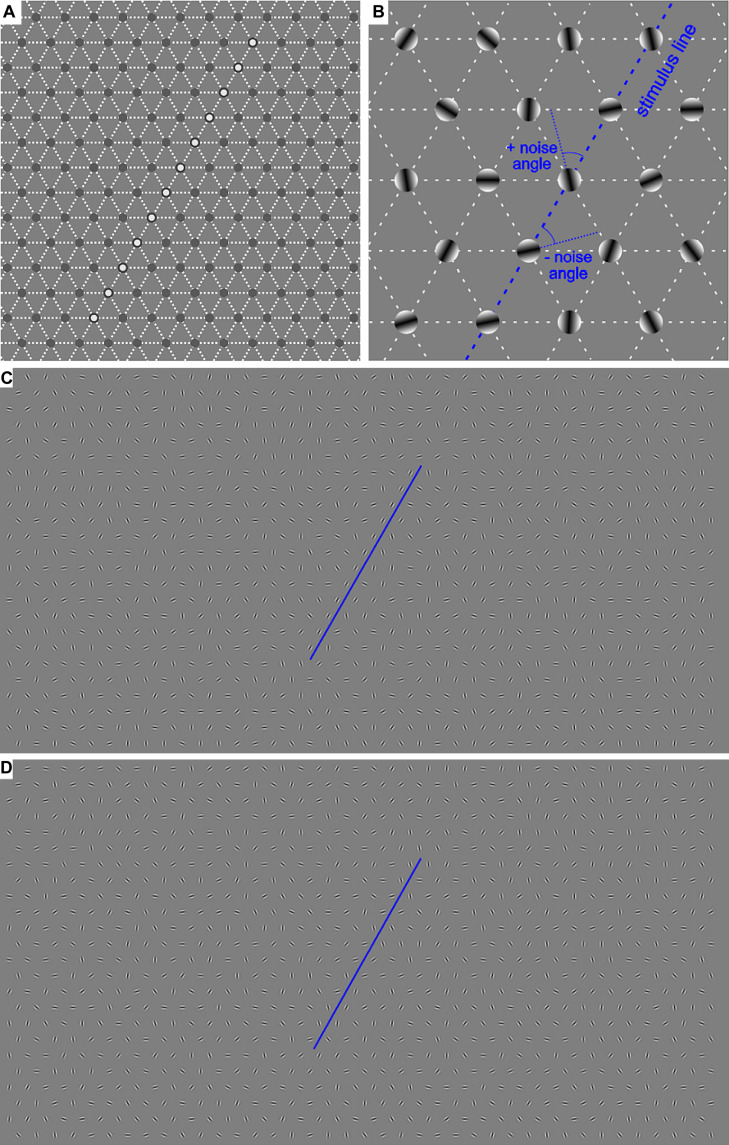
**(A)** Example of the central area of the invisible hexagonal grid on which the Gabor patches were positioned. The white circles in the most central area denote the positions where the contour appeared in half of the trials. **(B)** Demonstration of the generation of the external noise added to the contour. **(C,D)** Examples of the whole screen with a contour in the no-noise condition (0°, **C**) and in 45° noise condition **(D)**. The blue lines underline the contour stimulus.

A virtual contour (the target) of Gabor patches was embedded among similar patches with random orientation in the range of ±90°. The Gabor patches were positioned on a gray background at the intersection points of an invisible hexagonal grid of 39 columns × 25 rows. In such a way, 975 Gabor elements were generated and spaced at 1.044° (Figure 1). The Gabor stimuli had a spatial frequency of 5.75 cpd, a standard deviation of 0.087°, and a diameter of 0.522° with elongation 1.0 from a viewing distance of 70 cm. All Gabors were displayed at 75% Michelson contrast to avoid non-linear distortion of the monitor at very low and very high intensity. The average brightness of the stimuli coincided with that of the background. In half of the trials, the target contour consisting of 12 Gabor elements with the mean orientation of 60° was presented at the middle of the screen, as shown in Figure 1A. In the no-noise condition, all of the Gabor elements have a 60° orientation coinciding with the contour tilt. The external noise was defined as the orientation jitter added to the contour elements in the no-noise condition. Six noise levels (determined on a base of pilot experiments) were generated by adding or subtracting 0 (no-noise condition), 10, 20, 30, 45, or 60° to the orientation of the Gabor elements forming the contour. This manipulation preserves the mean orientation of the contour at 60°, but changes the variance of the contour elements; it is approximately equal to half of the maximal orientation change. The mean orientation of the rest stimulus elements was close to 0° with a standard deviation of about 50°. In the other half of the trials (non-target condition) the target contour was replaced by randomly oriented elements. The target or non-target stimuli were presented for 200 ms.

The precise parameters of the stimulation, such as the stimulus duration and noise levels were chosen based on pilot experiments in order to find the most suitable values for obtaining perceptual performance above the guess level and below 100%. A group of children and adolescents (6–16 years old) took part in the pilot experiments. We selected the method of constant stimuli as, if we have used an adaptive procedure, we would not be able to compare the performance of the participants in identical conditions; we would obtain only one value–the threshold representing the contour degradation the observers could tolerate, but we would miss the information about the participants’ sensitivity to the contour presence when no noise is added to the contour or at high noise levels.

The Yes/No procedure was used. The observers’ task was to indicate “as accurate and as fast as possible” (with the emphasis on the accuracy) the target presence or absence by pressing appropriate predetermined buttons on a controller. The six noise levels of the contour Gabor elements were separated into different experimental blocks. The separation of the stimuli in blocks reduces stimulus uncertainty and gives the participants the opportunity to adjust their perceptual template to the stimulus variability. Each block included 60 randomly ordered trials: 30 trials of target condition containing the contour and 30 trials with the non-target condition without a contour. The first trial was initiated by the participant pressing any button. Each next trial was triggered by the participant’s response to the previous trial. After an intertrial interval of 2000 ms the new trial started with the appearance of a blank gray screen of mean luminance with a fixation dot in the center accompanied by a warning beep. After a fore-period that varied between 500 and 1000 ms, the blank screen was replaced by target or non-target stimulus. The participants were instructed to look at the fixation dot, which coincides with the center of the target contour stimulus if it appears. Each experimental block started with six training trials: three trials contained target stimuli and three – non-target stimuli, the responses to which were disregarded.

Before the start of the first experimental block, stimuli at all noise levels with an unlimited stimulus duration were demonstrated to each observer and at least 1 training session at different noise levels was performed. Participants were given self-timed breaks between the separate blocks.

During each experimental trial, the gaze positions of the observer were recorded by the Gaze tracker Gazepoint GP3HD Desktop. The spatial accuracy of the eye tracker is 0.5–1°, and the resolution was set at 150 frames per second. The calibration was done with nine points of fixation and was checked with 11 points. If the check was not of good quality, the calibration was repeated.

The participants were in a darkened room without direct sunlight. The viewing was binocular, at a distance to the screen of 70 cm. The viewing distance of 70 cm was ensured by the fixed distance between the table under the monitor and the participant’s chair. The distance was verified periodically by using the gaze tracker control. Participant responses were collected via color-coded keys on a joystick controller. The responses, including the reaction time (RT), were processed by a custom device and transmitted to a computer.

In addition, a double pass paradigm was employed to assess internal noise (Burgess and Colborne, 1988; Vilidaite et al., 2017). Experimental blocks at two noise levels: low, 10°, and high, 45°, were repeated twice (two passes) by each participant in different daily sessions. The first pass was run in a predetermined random order, followed by the second pass with an identical stimulus presentation order.

Thus, each participant performed eight blocks altogether: six blocks at the six noise levels and two additional blocks repeated at the noise levels of 10 and 45°. The blocks of different noise levels were run in random order. The additional two blocks of 10 and 45° were always run last. In order to minimize tiredness, the data was obtained in 2 or 3 sessions of 2–4 blocks of trials, measured on different days.

After the procedures were fully explained (the details of the project and a participant information sheet), the parents provided informed written consent before inclusion. Informed consent was obtained orally from each participant. The decision regarding participation in the project was entirely voluntary. Participants received a voucher as a reward for participation. A researcher emphasized to the participants that their consent could be withdrawn at any time without penalty or affecting the quality or quantity of their medical/social or educational care, or loss of benefits to which the participant was otherwise entitled. One copy of the informed consent form was kept by the participant’s parents, while the other was kept by the research team. The experimental procedure was in accordance with the ethical standards of the Declaration of Helsinki and its later amendments or comparable ethical standards and was approved by the Ethics Committee of the Institute of Neurobiology, Bulgarian Academy of Sciences. All participants were cooperative and understood the task, as demonstrated by their performance in training trials.

### Statistical Analyses

All analyses included in the paper were performed in the R environment (R Development Core Team, 2014).

To compare the processes of contour detection performance in the two groups with different development, we used the bayesboot package (Baath, 2018) on the proportion of correct responses and the reaction time. The analysis allowed to estimate the confidence limits of these two characteristics of the performance using the values corresponding to 2.5 and 97.5% of the posterior distributions at each noise value. The default sample size of 4000 values was used. The probability of significant differences between the two groups at each noise value was also estimated. For the reaction time, we excluded all response times that were less than 0.25 s and more than 4.0 s as outliers.

To analyze the effect of noise level and the group on proportion correct responses, we use the lme4 package (Bates et al., 2015) for fitting a generalized linear mixed model regression for the binomial family with a logit link. In the analysis, we also used the IQ scores as a continuous predictor.

Also, we evaluated the relationship between the accuracy and the consistency of the responses in the double-pass of the experimental conditions at noise values of 10° and 45°. We used the methodology of Gold et al. (2004) to evaluate the ratio of the internal to the external noise σi/σe. This ratio was estimated from the following equation:

(1) $${\text{p}}_{c}\;=\;\text{m}*\text{log1}0\left({\text{p}}_{a}/\text{1}00\right)\;+\;\text{1}00\;\left(\text{1}\right)$$

In Eq. 1, p_*c*_ is the percent of correct responses, p_*a*_ – the percept of agreement between the responses from the two passes of the experiment and the slope m represents the ratio of the internal to the external noise. We used the nlme package (Pinheiro et al., 2020) to evaluate the two different values of noise for the two groups and the package emmeans (Lenth, 2019)–to evaluate whether the slopes differed. As we used two different values of external noise, one low and one high, the difference in the slopes will indicate whether the internal noise is additive or stimulus-dependent (multiplicative).

To analyze the effect of the group and the added external noise on the response time, we applied a generalized linear mixed regression model using the glmmTMB package (Brooks et al., 2017). We used Gamma distribution with an “identity” link function, as suggested by Lo and Andrews (2015). We also included in the analysis the IQ scores to evaluate the potential role of the intellectual abilities on response time.

In addition, the responses were separated into four categories according to the Signal Detection Theory (Green and Swets, 1966): hit (signal present and subject says “yes”), miss (signal present and subject says “no”), false alarm (signal absent and subject says “yes”), and correct rejection (signal absent and subject says “no”). The data in the different categories were used to verify the effect of the group and noise on the average percentage of the different response types for each participant at the different noise levels.

For the eye positions of the participants, we used spatial point pattern analysis (Baddeley et al., 2016). The mean coordinates of gaze positions for each trial and their standard deviations were estimated. We considered the distribution of gaze positions as spatial point patterns. We included in the analyses only the gaze positions allocated inside the presented image (i.e., inside the screen). As a result of this choice, 10% of the data of participants with autism and 4% of the data of participants with typical development were excluded from consideration. To compare the effects of noise and the differences between the two groups with different development, we used tools from the spatstat package (Baddeley and Turner, 2005). As the point patterns were generated by the eye positions from different trials, we considered them as independent and hence, as generated by a Poisson point process. A homogeneous distribution for a Poisson point process would imply complete spatial randomness. To evaluate whether the gaze positions are evenly distributed or clustered, we used the quadrat test. We also checked whether there was a difference between the distributions of the eye positions in trials when the contour was present (signal trials) and in the trials when only noise elements were presented (noise trials). For this purpose, we marked the points in the pattern depending on the type of stimuli (signal or noise) and applied a model of inhomogeneous Poisson distribution to the data. We used a second-order polynomial to describe the intensity (the expected density of points per unit area) of the distribution of the points as a function of their spatial coordinates. This choice implies the assumption that the gaze positions will be distributed in an elliptical region. The ppm function was used. This function is analogous to fitting a linear or generalized linear model to the point patterns.

To evaluate the contribution of the individual differences in each group on the variability of gaze positions, we used the pairdist function that gave the distance between all pairs of points in a pattern and estimated the summary statistics of the distances for different noise levels and groups. To determine whether each observer fixated the same locations on the screen for each noise level, we estimated the standard deviations of gaze positions in the repeated presentation of stimuli with the same added noise for each participant. We compared the differences in their distributions for each noise level using the bayesboot package (Baath, 2018).

## Results

In the present study, we recorded three types of performance characteristics: the response to the presence of contour, the response time, and the gaze of the participants. Below we present sequentially the analyses of these characteristics aiming to answer the question of whether the added external noise to a contour affects differently the detection performance of the two groups with different development.

### Effect of the Added External Noise on Sensitivity

**FIGURE 2:**
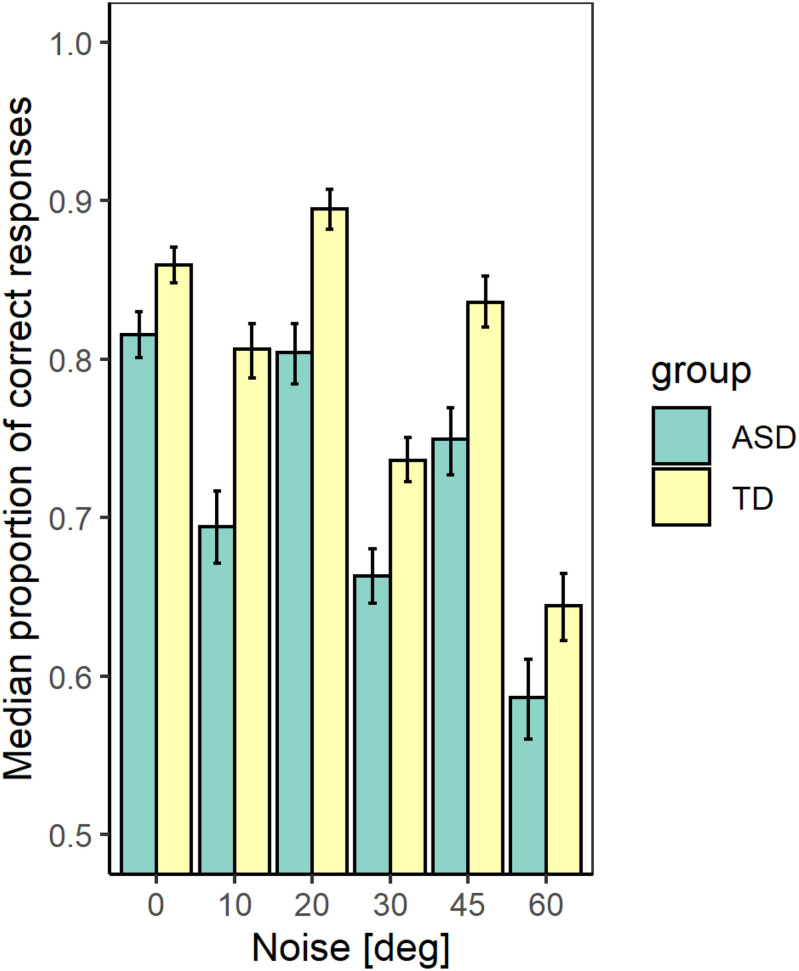
The median values of the proportion correct responses for the two groups with different development at different noise levels. The error bars correspond to the 2.5 and 97.5% values of a bootstrap sample of size 4000.

Figure 2 represents the median values of the correct responses of the participants from each group and noise level with confidence limits obtained from Bayesian bootstrap. The figure shows that at all noise levels the participants from the TD group achieved higher accuracy than the participants from the ASD group. We estimated the correlation between the mean proportion of correct responses and the IQ scores. The results show a significant positive correlation (*r*(54) = 0.63 [0.44 −0.77]; *p* < 0.001), implying that the detection performance depends on the intellectual abilities of the observers. To evaluate whether the group differences affect the performance irrespective of the intellectual abilities, we performed a generalized mixed model regression on the proportion correct responses, including as continuous predictors the noise level and the IQ scores and the group as a between-group factor. A random intercept and slope were included. The results show a significant effect of the noise level (χ^2^(1) = 179.04, *p* < 0.001), of the group (χ^2^(1) = 6.94; *p* < 0.01), and the FSIQ (χ^2^(1) = 4.25; *p* < 0.05). The interaction between the noise level and the group is non-significant (χ^2^(1) = 1.77; *p* = 0.18). The results show that the accuracy of contour detection evaluated by the proportion of correct responses decreases with the increase of the added external noise and increases with the IQ of the participants.

We verified the effect of the group and noise on the average percentage of the different response types for each participant at the different noise levels. The responses were separated into four categories according to the Signal Detection Theory (Green and Swets, 1966): hit, miss, false alarm, and correct rejection. The results show that both the group and noise have a significant effect on the number of hits, but their interaction is non-significant (χ^2^(1) = 15.69; *p* < 0.01 for the effect of the group; χ^2^(1) = 117.79; *p* < 0.01–for the noise effect, and χ^2^ (1) = 1.62; *p* = 0.20–for their interaction). Only the effect of the noise is significant for the number of false alarms (χ^2^(1) = 0.02; *p* = 0.98 for the effect of the group; χ^2^(1) = 70.85; *p* < 0.01–for the noise effect, and χ^2^(1) = 2.30; *p* = 0.13–for their interaction) as well as for the number of correct rejections (χ^2^(1) = 0.79; *p* = 0.37 for the effect of the group; χ^2^(1) = 165.51; *p* < 0.01–for the noise effect, and χ^2^(1) = 0.58; *p* = 0.44–for their interaction). For the number of misses, all effects are significant (χ^2^(1) = 14.53; *p* < 0.01 for the effect of group; χ^2^(1) = 165.51; *p* < 0.01–for the noise effect, and χ^2^(1) = 5.33; *p* < 0.05–for their interaction). The proportion of misses is lower for the TD group at all noise levels, but it increases more strongly with the noise increase for this group than for the ASD group. Sensitivity to contour detection depends on the proportion of hits and false alarms. Hence, our data imply an inferior ability to detect the contour presence for the ASD group. The deteriorated ability of contour detection is also supported by the higher proportion of misses for this group.

Using Eq. 1 and the data of the double-pass experiment, we obtained that the ratio of the internal to external noise m is 0.82 [0.77 −0.88] and 0.86 [0.81 −0.90] for the ASD and the TD group, respectively at noise level 10° and 0.67 [0.61 −0.72], 0.74 [0.69 −0.78]-for the two groups at noise value 45°. The values in brackets give the 2.5 and 97.5% lower and upper confidence limits. For both groups, the slopes at 10° and 45° differ significantly (*p* < 0.001). The difference in slopes between the two groups is non-significant at *p* = 0.05 for noise level 10° and differs significantly at a noise level of 45° (*t*-ratio = 3.58, *p* = 0.04). These results suggest that the internal noise for the two groups depends on the level of the added external noise, and the group of ASD participants is more affected by the increase in external noise. As the level of external noise for the two groups is the same, the lower slope for the ASD group implies either higher stimulus-dependent noise for this group or a suboptimal perceptual template. The non-significant difference of the slopes at the lower level of external noise suggests similar additive internal noise levels–the major limiting performance factor at low external noise levels.

**FIGURE 3:**
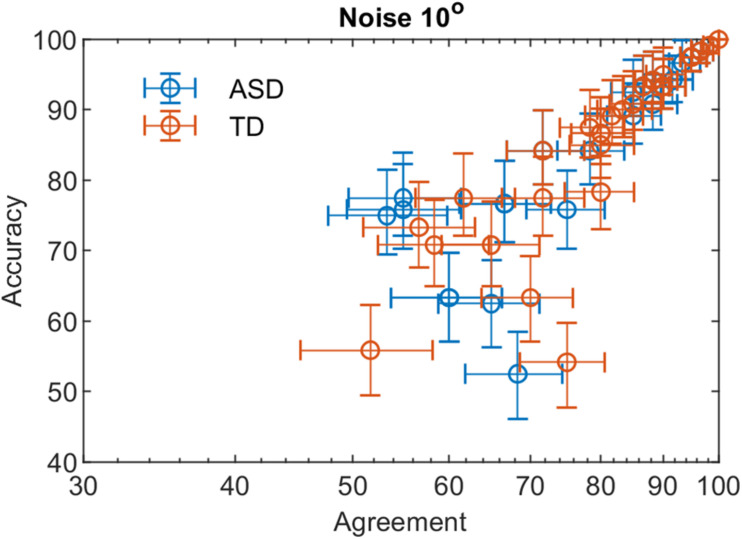
The percent correct responses and the proportion of agreement in a double-pass experiment at 10° added external noise for the two groups with different development.

**FIGURE 4:**
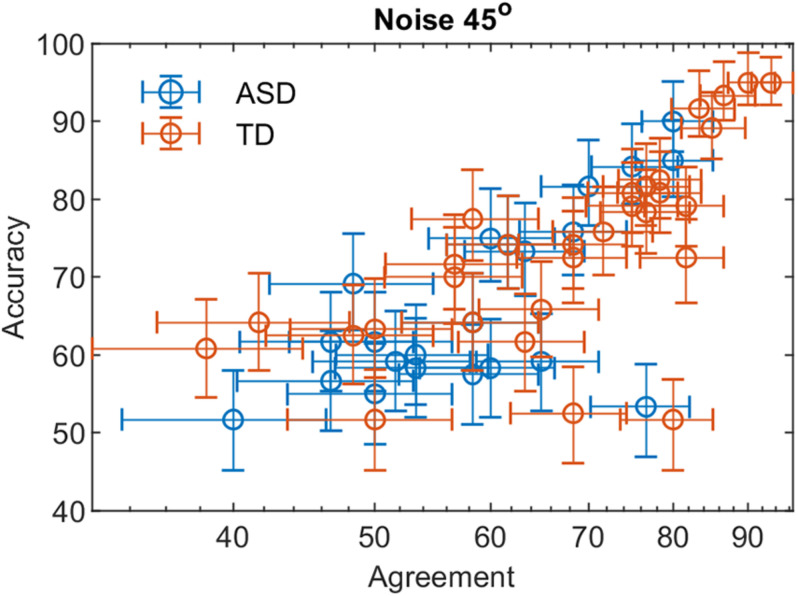
The percent correct responses and the proportion of agreement in a double-pass experiment at 45° added external noise for the two groups with different development.

Figures 3, 4 show the dependence of the percent correct responses on the proportion of agreement for the two groups for the two noise levels.

### Effect of the Added External Noise on Reaction Time

**FIGURE 5:**
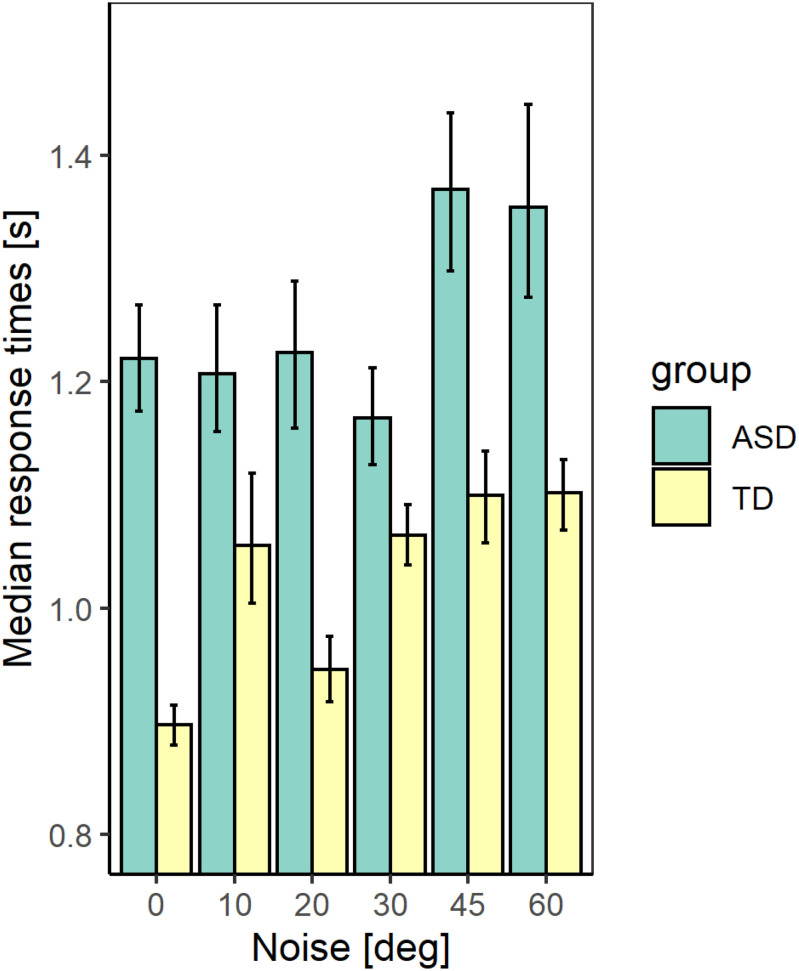
The median values of the response time for the two groups with different development at different noise levels. The error bars correspond to the 2.5 and 97.5% values of a bootstrap sample of size 4000.

**FIGURE 6:**
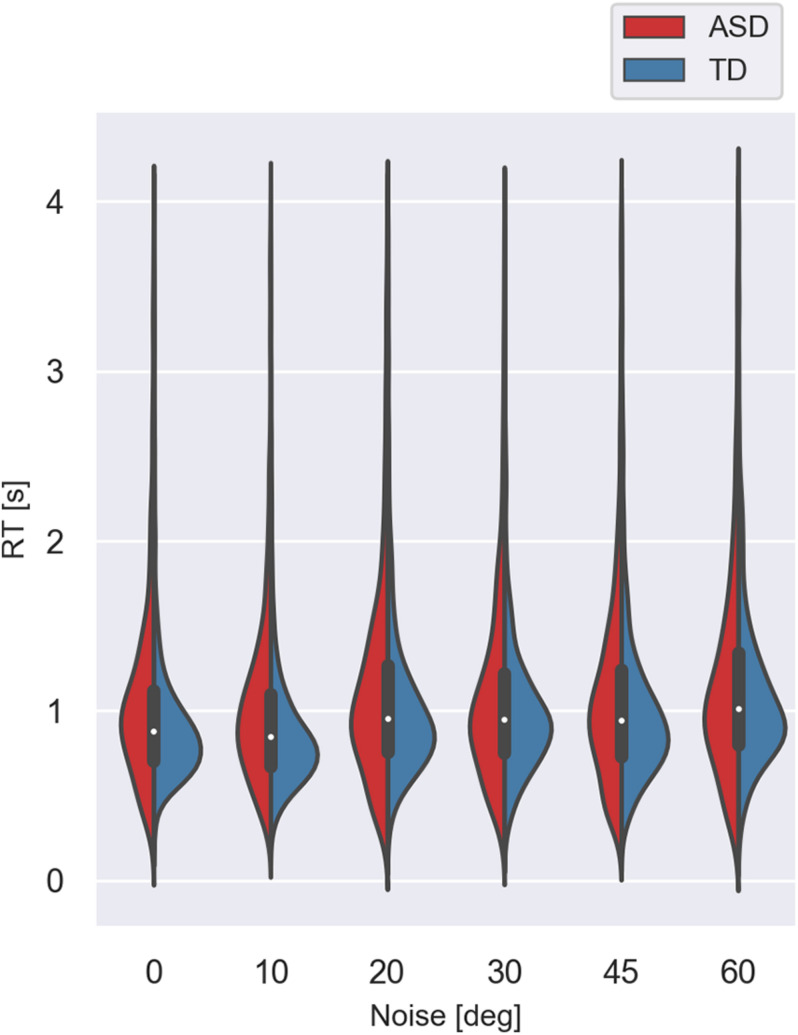
Violin plot of the variance of response times for the two groups with different development at different noise levels. The values lower than 0.25 s and higher than 4.0 s were excluded.

Figures 5, 6 show the median values of the RT for the participants of the two groups and the variability of its values at each noise level obtained using Bayesian bootstrap. The results imply that in all conditions, the RT for the ASD group is longer than the RT of the TD group and has a higher variability. For both groups, the increase in the external noise leads to an increase in the RT and its variability. The response time reflects different cognitive processes, some of them related to decision-making, others–to the encoding of the stimulus information and the motor response’s preparation and execution (Ratcliff and McKoon, 2008). For example, the response time may increase due to the observers’ attempt to keep high accuracy and hence, needing more evidence before making a choice. It could also depend on the task difficulty that affects the rate of evidence accumulation. We estimated the correlation between the mean response time and the IQ scores to evaluate whether the observers’ intellectual abilities affect their response time. The results show non-significant correlation between these two characteristics (*r*(54) = −0.24 [−0.47 −0.03], *p* = 0.07. This outcome suggests that the differences between the two groups observed in Figure 5 might be related to stimulus encoding and motor response preparation processes.

We performed a generalized mixed linear model on the response time using as continuous predictors the levels of added external noise and the IQ scores and the group as a between-subjects factor. We included random slopes and intercepts to account for the individual differences. The results show significant effects of the noise level (χ^2^(1) = 24.04; *p* < 0.001) and the group (χ^2^(1) = 8.11; *p* <, 01). The effect of the IQ score (χ^2^(1) = 0.01; *p* = 0.92) and the interaction between the noise level and the group (χ^2^(1) = 1.69, *p* = 0.19) are non-significant. The increase in the noise level leads to a prolongation of the response time that could reflect a change in the task’s difficulty with increased noise. The outcome of the analysis, however, implies that even though the ASD group responds more slowly, the noise affects their response time similarly to the TD group.

### Effects of the Added External Noise on Gaze Allocation During Stimulus Presentation

The experiment was conducted with the presentation of a fixation point located in the center of the stimulus. In the trials, when the contour was presented it always appeared in the same location. Therefore, it can be assumed that the participants will maintain a stable fixation during the stimulus duration because it was only 200 ms. For this reason, it can be expected that there will be no difference in the distribution of the gaze positions between the different experimental blocks, corresponding to different levels of added noise to the contour, as well as between the two groups with different development. However, if the fixation stability differed between the two groups, a difference between the distributions of gaze positions might occur. Still, no difference between the blocks with different noise levels is expected. A third potential scenario is that the participants direct in advance their gaze to the parts of the image that they expect to carry the most relevant information about a contour’s presence. The redirection of the gaze is carried out during the presentation of the fixation point. In this scenario, a different distribution of gaze positions may be expected depending on the participants’ group and the amount of noise added to the contour.

First, we evaluated whether the gaze allocations were evenly distributed or are clustered. The analysis showed that for all experimental conditions and all groups studied, the eye positions were clustered, and their distributions are inhomogeneous (quadrat count test, χ^2^(24) = 21630 and 22164 for children with ASD for the signal and noise trials, respectively, and 149924 and 151170 for children with TD in these conditions, *p* < 0.001). The graphical comparison of the envelope of Ripley’s K-function showed that the gaze positions form point patterns that are not only inhomogeneous but also that for the autistic group the distance between the points is greater than expected based on the estimated intensity function for non-homogeneous patterns.

We tested whether the distributions of the gaze positions for the signal and noise trials differ. We fitted an inhomogeneous Poisson point process model on the intensity of the point patterns as a second-order polynomial function of their spatial coordinates separately for each group. This analysis showed that for both groups, the distribution of eye positions did not depend on the presence or absence of a contour (χ^2^(1) = 0.73; *p* = 0.39 for children with ASD and 0.23; *p* = 0.69 for children with TD). The lack of difference between the signal and noise trials is expected as the observers could not know the type of the presented stimulus in advance. It may be due to maintaining constant fixation or to the use of the “history” of the presented stimuli to predict the most informative parts of the images in determining the presence of a contour. To distinguish between these two hypotheses, we created a hyperframe, i.e., a data frame that contains objects of any kind. We included in the hyperframe the point patterns obtained for stimuli with different levels of added noise for the two groups of participants and tested the effect of these factors on the gaze allocation. Here again, we assumed that the intensity of the point patterns depends on the spatial coordinates of the points as a second-order polynomial. The point patterns were considered as samples from inhomogeneous Poisson distribution. The results show a significant effect of the noise level (χ^2^(5) = 967.54; *p* < 0.001) and of the group (χ^2^(1) = 2321.30; *p* < 0.0001), as well as a significant interaction between the level of the added noise and the group (χ^2^(5) = 206.82; *p* < 0.001). There are also significant effects of the spatial coordinates (χ^2^(5) = 13409.29; *p* < 0.001 for the combined effect of the 5 elements of the second-order polynomial: x, y, x^2^, y^2^, and x^∗^y), of the interaction between the noise level and the spatial coordinates (χ^2^(25) = 1559.46; *p* < 0.001), and of the interaction between the group and the spatial coordinates (χ^2^(5) = 2539.59; *p* < 0.001). The triple interaction between the spatial coordinates, the noise level, and the group is also significant (χ^2^(25) = 2123.80; *p* < 0.001). The effect of noise on the distribution of gaze positions implies that the participant might be using the “history” of stimulus presentation to locate the contour.

The comparison of the distribution of the pattern intensity indicates that the position of the pattern in horizontal (x) and vertical direction (y) differs at all noise levels except at noise level 0° (no added noise). The intensity of the gaze point patterns does not differ significantly in vertical direction also at noise level 45°. At all other noise levels, the distribution of gaze positions differed in x^2^, x^∗^y, and y^2^ implying different elongation and spread of the gaze positions. Whereas the effect of the group on the pattern intensity might be due to the higher number of gaze records for the typical children in the screen area, the interaction between the group, the spatial coordinates, and the noise level implies that the children from the two groups allocate their gaze to different portions of the image at the different noise levels.

We also calculated the variance ellipses of the gaze positions at different noise levels for the two groups. We estimated first whether the variance ellipses could be regarded as elongated, i.e., whether there is a significant difference between the maximal and the minimal variance of the distributions. In all cases, the *F*-test for variance comparison suggests that the distributions could be regarded as elongated (*F* = 2.18, 2.25, 2.36, 3.26, 2.66, 3.33–for the ASD group and 2.78, 2.61, 1.99, 3.18, 1.66, 2.69–for the TD group; *p* < 0.01). We also compared separately the maximal and the minimal variance of the gaze positions for each noise level for the two groups. For all noise levels, the maximum variance was greater for children with ASD than for the TD group (*F*-test: 1.64, 3.42, 3.02, 1.75, 2.32, 4.29 for noises from 0 to 60°, *p* < 0.01). Also, the minimal variance of the distributions for the children with ASD significantly exceeded those for children with TD (*F*-test: 2.09, 3.96, 2.55, 1.71, 1.44, 3.47; *p* < 0.01). These results suggest greater variability of eye positions for children with ASD that may be due to decreased fixation stability or larger individual differences in the selection of the most informative sections of images at different noise levels.

To discriminate between these two possibilities, we calculated the mean gaze positions for each participant and created a new point pattern using the two groups as marks. We estimated the distance between all pairs of points for each group. If the mean gaze positions of the different participants are closer, the distance between them will be smaller than if they are more dispersed. Therefore, the distribution of the distances between the mean gaze positions of any two group members could be used to measure the individual differences in this group. The median value obtained for the distances between the mean gaze positions of each pair of participants in the ASD group is 0.203 [0.110 −0.339], and it exceeds the median value of 0.124[0.059 −0.0240] for the TD group significantly (the values in brackets are for the first and the third quartiles). These results suggest more considerable individual differences in mean gaze positions of the ASD group than in the TD group.

These results may imply that the effect of noise has a different impact in the ASD group, increasing the dispersion of the mean gaze positions or that at each level of noise, the individual differences of the eye positions for this group are larger than for the TD group. The comparison of the median values of the paired distances for each noise value implies higher variability for the ASD group.

We also use Bayesian bootstrap to evaluate the differences in the variability of the gaze positions of the members of each group at a different noise level. This measure would indicate whether each participant fixated the same screen position in the block of trials with the same external noise. The results suggest a higher variability of gaze positions in the ASD group than in the TD group for noise levels of 20°, 45°, and 60°. The higher variability of gaze positions for the ASD group in comparison to the TD group at 45° and the non-significant difference at 10° noise level may be interpreted as an indication that the higher ratio between the internal and the external noise obtained from the double-pass experiment may be caused by the higher variability of the gaze positions in the ASD group. Since the variability is stimulus-dependent, it might suggest an unstable, noisy, or suboptimal perceptual template of the ASD group. The improper template would lead either to the omission of important stimulus information or the inclusion of irrelevant features and hence, to reduced ability to filter external noise.

**FIGURE 7:**
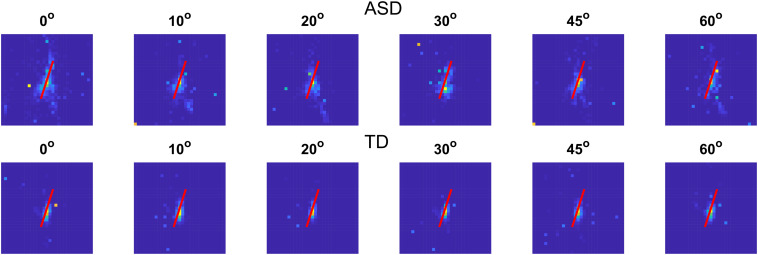
Histogram of the gaze locations on a 31 × 31 grid for the two groups with different development at different noise levels. Gaze positions outside of the screen were excluded. The red line shows the contour position.

Figure 7 shows a histogram of the gaze positions on a 31 × 31 grid in the screen window for each group at each noise value.

We also estimated the correlation between the area of gaze positions and the proportion of correct responses and the response time to test whether the fixation instability could cause a deterioration in task performance. The correlation coefficients are significant (*r*(54) = −0.45 [−0.66 to −0.18], *p* < 0.01 for the proportion of correct responses and 0.33 [0.08 − 0.55], *p* < 0.05 for the response time). The values in brackets show the 95% confidence intervals. The significant correlations imply that when the gaze is spread over a larger area, the observers are less accurate in detecting the contour and need more time to make a choice.

To test whether the intellectual abilities affect the spread of the gaze distributions, we estimated the correlation between the area of gaze positions and the IQ score. The significant negative correlation of *r*(54) = −0.34 [−0.55 to −0.09], *p* < 0.05, indicates that children with lower intellectual abilities have more dispersed gaze positions.

In summary, the analyses of the gaze positions show significant differences between the two groups with different development depending on the noise level added to the contour embedded in the background noise. These findings could be interpreted as an indication that the two groups have a different choice of which portion of the image is more informative for the presence of a contour and that this choice depends on the level of added noise. Also, the gaze positions of the children with ASD are more dispersed, implying greater individual differences and greater instability in fixations.

## Discussion

The results obtained in the present study showed atypical contour integration processing in autism, probably due to difficulties in rejecting background noise and integrating the elements of a jagged contour. The responses of the group with ASD were less accurate and significantly slower compared to the control group, even in the no-noise condition. In line with the psychophysical data, the eye-tracking results showed a larger gaze allocation area in the ASD. Our findings also indicate that the response time changes with the level of added external noise similarly for the two groups with different development remaining longer for the ASD group. The dependence of the response time on the stimulus characteristics suggests that either the rate of evidence accumulation (the component of response time that depends on task difficulty) or the time needed to encode the stimulus characteristics increases with the noise level increase. It also implies that the response time prolongation in the ASD group may be predominantly due to factors related to the motor response preparation and execution. The external noise added to the contour had a larger effect on gaze positions of ASD participants inducing larger dispersion of the mean gaze positions and higher variability in the ASD group. The significant correlation between the area of the gaze positions of each participant and the mean proportion of correct responses and the mean response time implies that the area of gaze positions affects children’s ability to detect the contour. The comparison of the agreement and accuracy of the responses in the double-pass experiment showed that the participants with ASD are more affected by the increase of the external noise. It turned out that the internal noise depends on the level of the added external noise: the difference between the two groups was non-significant at the low external noise and significant at the high external noise.

There are many differences between our research and the previous studies investigating contour integration in ASD individuals, like the experimental procedure, the sample size, the choice, and the characteristics of the participants. We will first discuss the potential effect of these differences before focusing on our study’s main distinguishable feature: contour position and noise manipulation.

### Effect of the Experimental Procedure and Contour Characteristics

Since the pioneering work of Field et al. (1993) in contour integration studies, including those that involve participants with ASD, the forced-choice procedure is the most explored approach, being a temporal two-interval forced-choice (e.g., Jachim et al., 2015) or spatial four-alternative forced-choice (e.g., Del Viva et al., 2006). Although we used open contours and a detection task, our data are in line with studies that show diminished contour integration in the ASD group (Pei et al., 2009; Evers et al., 2014; Jachim et al., 2015). In fact, the performance of ASD participants was not diminished in the open contour integration task in Jachim et al. (2015), probably because of the small group size as suggested by the authors. However, the benefit from the closed contours was reduced in the ASD group, which led the authors to conclude weaker contour integration in adults with ASD. Gowen et al. (2020) replicated the findings of Jachim et al. (2015) about the reduced closure effect in autistic individuals with a new larger group of participants with ASD. However, in contrast to the first study, the result from the newest study (Gowen et al., 2020) found differences for the open stimulus between groups with ASD and TD: the perceptual performance was even better for the autistic than for the non-autistic group.

Probably, the number of contour elements could reduce the contour integration ability of our ASD group. The number of contour elements used in the present study is lower (12 Gabor patches) in contrast to the many more elements, 20 and 35, that constructed the contours, respectively in studies of Jachim et al. (2015) and Gowen et al. (2020). More elements could enhance autistic performance as the comparison of the results from the works mentioned above shows. This assumption is also supported by neurophysiological results that contour detectability improved with the increase in the number of collinear line elements (Li and Gilbert, 2002).

### Effect of Sample Size and Individual Characteristics

The groups of participants with ASD in Jachim et al. (2015) and Gowen et al. (2020) were smaller, more compact, and homogenous (the samples included only participants with a diagnosis of Asperger’s syndrome) than our group with ASD. Moreover, in contrast to our group with ASD, participants in their studies were adults, 18–42 years old. This could also influence the results because there is a prolonged development for contour processing, as suggested by Taylor et al. (2014). The sample size and the age range of participants in the study of Evers et al. (2014) have several similarities with our cohort. The age range of the children and adolescents was similar to ours: 10–17 years old compared to 8–16 years old in our study. They used ADOS to confirm the diagnosis and to measure the severity of the ASD symptoms. The participants’ scores ranged from 2 to 9 or from 3 to 10 (severity scores), 4–5 scores (ASD-classification), and 6–10 scores (receiving an autism classification) (Gotham et al., 2009). In such a way, some children are outside of the ranges for ASD, again raising the question of severity measurement. The group with ASD in Evers et al. (2014) was as large as our group and they found diminished identification performance of the children with ASD (see the severity score range). However, the task in their study was more complicated and it is not clear if the results are due to the larger group or a more complex task.

Because ASD is a complex, pervasive, highly heterogeneous condition with multiple subtypes and developmental trajectories, the size of the group and the choice of participants included in the study could also influence the results obtained. In order to encompass as many as possible cases from the autism spectrum, we tried to include a large sample representative for the heterogeneity of the disorder, where participants with ASD were not excluded based on their cognitive level functioning as could be seen in Table 1. The FSIQ score ranged from 59 to 122 in the ASD group and was significantly different from the FSIQ score of the TD group. Other scores, VCI, WMI, and PSI, also differed significantly for both groups of participants. Curiously, the PRI score, which could be presumably the most related to the performance of the visual task in our study, did not differ significantly between the ASD and TD groups. In addition to the IQ scores, Gowen et al. (2020) discussed that the autism severity could be connected to contour integration results through variability in the integrity of lateral interactions (as suggested by Dickinson et al., 2018). Using a steady-state visual evoked potential paradigm, they found that greater ASD symptom severity, assessed as an increased ADOS score, is associated with increased short-range lateral inhibition. The severity of the autistic disorder is a complicated topic, and the accurate assessment is still a challenge. DSM-5 includes a severity marker based on the degrees of impairment in the domains of social communication and restricted and repetitive behaviors. Although qualitative differences between impairment levels are described in the classification (DSM-5; American Psychiatric Association [APA], 2013), quantitative methods for differentiating between these levels are still a problem. Levels of impairment in children with ASD are usually associated with language delay, cognitive functioning, or behavioral issues such as aggression. Although these factors are important in the overall adaptive functioning, they are not core features of the autism spectrum. Notwithstanding that ADI-R could not assess directly the severity of symptoms, it should be noted that the mean group results in the present study in each of the three domains are high, and all participants in the experimental cohort are classified as patients with autism according to their scores from ADI-R since all they have results above the cut-off level in each of the three domains and exhibit some abnormality in at least one area by the age of 36 months.

**FIGURE 8:**
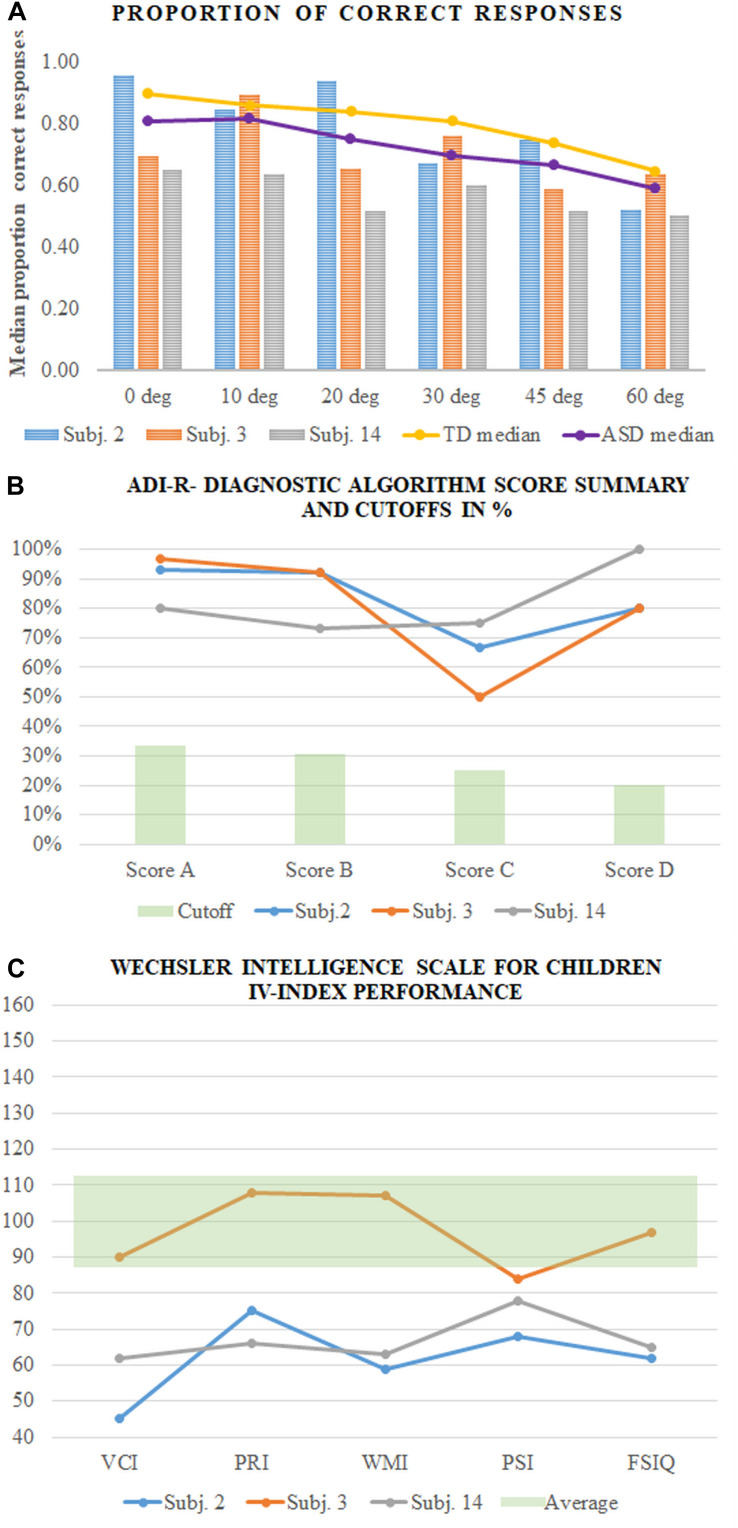
Results of the three participants (Subjs. 2, 3, and 14 in our sample). **(A)** The proportion of correct responses of each participant compared to the medians of the group with ASD and TD; **(B)** ADI-R- Diagnostic Algorithm Score Summary and Cutoffs in%; **(C)** Wechsler Intelligence Scale for Children IV-index performance.

Our results showed a relationship between the spread of the gaze positions, the proportion of correct responses, the response time, and the IQ scores. IQ scores also affect the accuracy of the task performance. The detection performance depended on the group differences in addition to the effect of the intellectual functioning of the participants from both groups. These results do not, however, represent the complicated picture for an individual. As an illustration of the relationship between the IQ scores, symptom severity, and contour integration performance, we decided to compare the data of the participants of the same age from our group with ASD. Moreover, this will allow capturing what Hodkinson and Hodkinson call “lived reality” (Hodkinson and Hodkinson, 2001) and to avoid the group results to absorb the individual ones. We found three male participants (Subjs. 2, 3, and 14) at approximately the same age (13.7, 14, and 13.7 years old). Figure 8 presents the results of the three participants: the proportion of correct responses (Figure 8A), ADI-R- Diagnostic Algorithm Score Summary and Cutoffs (Figure 8B) and VCI, PRI, WMI, and PSI scores assessment by WISC IV (Figure 8C). The figure clearly shows that the much better perceptual performance of Subj. 2 compared to Subjs. 3 and 14 could not be explained by the potential difference in any of the scores from the psychological assessments. Moreover, Figure 8A shows different individual dependencies of the proportion of correct responses on the external noise level. Noise increase has the strongest effect on the results of Subj. 2 despite his best results in the no-noise condition. This observation implies more complicated relationships between all of the discussed factors that need to be elucidated in further research. It also implies that the performance at a low or no-noise condition that is limited predominantly by additive internal noise cannot predict the performance at high noise levels that is limited by the ability of noise filtering and the efficiency of stimulus information exploration.

### Role of Internal Noise and Perceptual Efficiency in ASD

Several studies reported results that were interpreted as evidence against theories of reduced global perception in autism. Besides the already mentioned works of Del Viva et al. (2006) and Kemner et al. (2007), Gowen et al. (2020) also found similar contour integration performance with closed, simple shapes. In line with these findings, Zaidel et al. (2015) reported intact global and multisensory integration in ASD. At the same time, the authors found a specific sensitivity to dynamic visual noise in the participants with ASD. These results were interpreted as evidence against theories of reduced global perception in autism. Zaidel et al. (2015) assumed that increased sensitivity to noise rather than diminished integration ability is a distinguishing feature of ASD.

Dinstein et al. (2015) suggested that increased neural noise in sensory and motor systems may explain why individuals with ASD suffer from different problems that affect multiple aspects of day-to-day functioning: balance problems, motor clumsiness, atypical visual perception, and abnormally large behavioral variability in trial-to-trial reaction times, eye saccade accuracy, reaching movement accuracy, and pitch of voice during speech. Moreover, neural noise theory (Simmons et al., 2007) proposes that neural noise accounts for the complex pattern of enhancements and impairment in the ASD population (see also Simmons et al., 2008, 2009).

Park et al. (2017) tried to estimate quantitatively different sources of noise that limit perceptual processing in ASD. The authors applied an equivalent noise paradigm and modeled the individual visual orientation discrimination at variable levels of external noise. It was found that the high internal noise, as well as poor external noise filtering, restricts visual processing in ASD. However, the severity of ASD symptoms correlated significantly only with internal noise estimates.

The results from the double-pass experiment in our study imply either a higher stimulus-dependent noise for the group with ASD or a suboptimal perceptual template. The non-significant difference between the groups at the lower level of external noise suggests similar additive internal noise.

In our study, the contour and the noise elements were at the intersections of regular hexagonal grid lines. The centroid positions of all Gabor elements (noise and contour) were perfectly aligned. Hence the contour detection could not be based on the positional information, but only on differences in the mean element orientation and its variability along the grid lines. The mean orientation of the contour was fixed at 60°, and we varied the orientation variance of the elements forming the contour. The perceptual organization cues that could help to segregate the contour are good continuation and similarity, and it is shown in previous studies (Avraam et al., 2019) that ASD participants could use typical perceptual organization cues. However, good continuation is an effective cue only at low added noise. The similarity could be determined either by the orientation or the variance in the contour elements’ orientation. Hence, it is quite possible that the participants in our study changed their strategy depending on the external noise added to the contour. The change in strategy most probably depends on the sensitivity to the added external noise. We observed differences between the proportion of correct responses and the response consistency at low and high noise levels. One interpretation of this difference could be a change in strategy. The different spread of the gaze allocations at low and high noise could also indicate a noisier and unstable perceptual template at high noise levels. This finding raises the question of what information could the eye position measurements in our study provide.

The stimulus duration in our study is relatively short, and eye movements during the stimulus presentation could not affect the contour detection. However, we have a variable and relatively long fore-period before the stimulus appears. Our results showed significant differences in the spread of gaze positions between the two groups with different development. This finding indicates greater fixation instability for the ASD group. Few data exist on fixation stability in the age group used in the study and even less–for the group of children with ASD (Sumner et al., 2020). The study of Sumner et al. (2020) indicates that the ASD group keeps fixation for shorter times and has more intruding saccades than the TD group. However, differences between the ASD and TD groups disappear when the motor skills of the participants are taken into account. Our data provide additional knowledge about gaze characteristics of children and adolescents with ASD. Our results about the atypically larger gaze area in ASD are in line with previous results about abnormal eye control (e.g., Takarae et al., 2007) and could contribute to a better understanding of the deteriorated results on contour integration in the ASD group. They could also be considered as an indication of higher positional uncertainty in the group with ASD as compared to the TD group.

The different spread of the gaze positions in our study, however, is also related to the stimulus characteristics. It varies with the added external noise, suggesting that the gaze allocation is related to the external noise level. Due to the block stimulus presentation, the results may be interpreted as indicating that at high noise levels, the observers have difficulty determining the most informative parts of the stimulus, i.e., to have a proper perceptual template that will allow filtering the background noise and effectively using useful stimulus characteristics. The participants from both the ASD and TD groups could probably use the “history” of the presented stimuli to predict the most informative parts of the images about the contour presence, reflected in the lack of effect of the contour presence. The results of more dispersed gaze positions in the ASD group together with the stronger effect of the noise level are consistent with the assumption that individuals with ASD possess a stronger reliance on incoming sensory information and less use of prior knowledge about the world referred to as an attenuated Bayesian prior (Pellicano and Burr, 2012; Zaidel et al., 2015).

In conclusion, the results of the present study showed diminished contour integration ability in ASD, as the data were obtained from a sample that is representative of the disorder’s heterogeneity. The proportion of correct responses for the contour detection was lower while the proportion of misses was higher, and the time to respond was prolonged in the ASD group at all noise levels. These results could indicate difficulties for the ASD group to integrate the elements of a jagged contour. The deviation of the individual elements from the contour path, even at the highest noise level, is in the critical limits of the associate field if used to represent contour goodness (Field et al., 1993). However, the maximum path angle that could be detected depends on the background elements’ statistics (Watt et al., 2008). The deteriorated performance of the participants with ASD might be due to their inability to distinguish the target from the background noise. The comparison of the accuracy and agreement between the responses in the double-pass experiment showed that the performance of the participants with ASD is more affected by the external noise increase whilst the results of both groups were similar when external noise was low. The results obtained suggest reduced efficiency to use the available stimulus information of the participants with ASD. Also, the gaze positions of the ASD group were dispersed over an atypically large area. These findings imply lower efficiency in using stimulus information and higher positional uncertainty in the ASD group that could be caused by unstable fixation and poorer noise filtering.

## Data Availability Statement

The raw data supporting the conclusions of this article will be made available by the authors, without undue reservation.

## Ethics Statement

The studies involving human participants were reviewed and approved by Ethics Committee of the Institute of Neurobiology, Bulgarian Academy of Sciences. Written informed consent to participate in this study was provided by the participants’ legal guardian/next of kin.

## Author Contributions

MM, NB, and TT contributed to the study conception and design. All authors performed the material preparation, data collection, and analysis, wrote first draft of the manuscript, commented on previous versions of the manuscript, read and approved the final manuscript.

## Conflict of Interest

The authors declare that the research was conducted in the absence of any commercial or financial relationships that could be construed as a potential conflict of interest.
